# Therapeutic Potential of Dimethyl Fumarate in Counteract Oral Squamous Cell Carcinoma Progression by Modulating Apoptosis, Oxidative Stress and Epithelial–Mesenchymal Transition

**DOI:** 10.3390/ijms24032777

**Published:** 2023-02-01

**Authors:** Rossella Basilotta, Marika Lanza, Alessia Filippone, Giovanna Casili, Deborah Mannino, Federica De Gaetano, Giulia Chisari, Lorenzo Colarossi, Gianmarco Motta, Michela Campolo, Salvatore Cuzzocrea, Irene Paterniti, Emanuela Esposito

**Affiliations:** 1 Department of Chemical, Biological, Pharmaceutical and Environmental Sciences, University of Messina, Viale Ferdinando Stagno D’Alcontres 31, 98166 Messina, Italy; 2 Istituto Oncologico del Mediterraneo, Via Penninazzo 7, 95029 Viagrande, Italy

**Keywords:** OSCC, DMF, oncology, oxidative stress, apoptosis, EMT

## Abstract

Oral squamous cell carcinoma (OSCC) is a common human tumor, that originates from buccal mucosa and the tongue, associated with a high mortality rate. Currently, the treatment for OSCC involves surgery, chemotherapy and radiotherapy; however, survival outcomes for OSCC patients remain poor. For this reason, it is necessary to investigate new therapeutic strategies to counteract the progression of OSCC. In this study, we aimed to evaluate the role of dimethyl fumarate (DMF) in modulation of OSCC progression, both in vitro and in an in vivo orthotopic xenograft model. In vitro results revealed that DMF was able to reduce the expression of anti-apoptotic factors as BCL-2 and increased the expression of pro-apoptotic factors as Bax, Caspase-3 and BID. DMF appears to be involved in the modulation of oxidative stress mediators, such as MnSOD and HO-1. Furthermore, DMF showed to reduce the migratory ability of tumor cells and to modulate the expression of markers of epithelial-mesenchymal transition (EMT), as N-cadherin and E-cadherin. The in vivo study confirmed the data obtained in vitro significantly decreasing tumor mass and also reducing oxidative stress and apoptosis. Therefore, based on these results, the use of DMF could be considered a promising strategy to counteract oral cancer progression.

## 1. Introduction

Oral squamous cell carcinoma (OSCC) includes epithelial tumors that originate from the lip, oral cavity and lining of the oropharyngeal mucosa and is one of the most common malignancies of the oral cavity [1]. In particular, OSCC ranks among the top 10 tumors in developing countries with an annual incidence of 450,000 new cases and 230,000 deaths reported each year [2,3]. Despite innovative strategies to improve prevention and early diagnosis and recent advances in the treatment of OSCC, the 5-year survival rate still remains around 50% [4]. Oncological therapy for OSCC, which includes surgery, radiotherapy and chemoradiation, has often negative effects on the patient’s vital functions such as breathing, swallowing, speaking ability, physical appearance and, therefore, on the quality of life [5]. The identification of effective prognostic factors, which reliably predict the aggressive behavior of the tumor and the progression of the disease, can represent an important aid in the recognition of cases at higher risk and in the choice of therapeutic strategies to be adopted to improve the clinical outcome. A lot of evidence reveals that some lifestyle factors, including tobacco and alcohol use, but also sexually acquired human papilloma virus (HPV), are the causes of the vast majority of OSCC cases [6,7,8]. Exposure to these carcinogens is associated with a series of molecular and cellular events that culminate in neoplasm. In this context, free radicals and reactive oxygen species (ROS) play an important role in the pathogenesis of OSCC by causing DNA damage, responsible for initiating and promoting oral carcinogenesis [9]. Abnormal production of ROS can lead to oxidative stress which causes many pathophysiological alterations of normal signaling proteins, macromolecules and nucleic acids and facilitates the epithelial-mesenchymal transition (EMT), causing significant morphological changes from the epithelial to mesenchymal phenotype that promote greater progression and invasion [10].

Dimethyl fumarate (DMF) is a fumaric acid ester FDA-approved as a treatment for multiple sclerosis (MS) [11]. To date, the mechanism of action is still unclear. DMF is orally bioavailable and once absorbed is rapidly hydrolysed by esterases to monomethyl fumarate (MMF). MMF has a short half-life (36 h) and is able to cross the blood–brain barrier (BBB) and interact with immune cells present in the bloodstream [12]. DMF is involved in the reduction of oxidative stress by inducing multiple antioxidant factors mediated by the nuclear factor-erythroid 2 related factor 2 (NRF2) pathway [13]. Numerous studies have indicated that DMF exerts beneficial effects on a variety of cancers [14,15,16]. DMF was shown to reduce the release of ROS in macrophages by activating NRF2, thus weakening the invasion capacity of tumor cells in breast cancer patients [17]. DMF also showed a broad antitumor effect in diffuse large B-cell lymphoma (DLBCL), inducing ferroptosis and altering nuclear factor kappa–light-chain-enhancer of activated B cells / signal transducer and activator of transcription 3 (NF-κB/ STAT3) signaling pathway, leading to a form of cell death driven by phospholipid peroxidation [18]. This antitumor effect was further found in metastatic melanoma, where DMF reduced metastases and tumor growth by preventing nuclear translocation of NF-κB and inhibiting the expression of matrix metalloproteinases (MMPs), of Survivin and Bcl-extra-large proteins (Bcl-XL) [19]. Therefore, considering the numerous evidences regarding the anticancer properties of DMF, the aim of this study was to investigate the potential effect of DMF on the reduction of oral cancer growth in an in vitro and in vivo orthotopic model of OSCC, in order to consider its potential use as a new therapeutic strategy.

## 2. Results

### 2.1. In Vitro Results

#### 2.1.1. DMF Reduced OSCC Cell Viability

**Figure 1:**
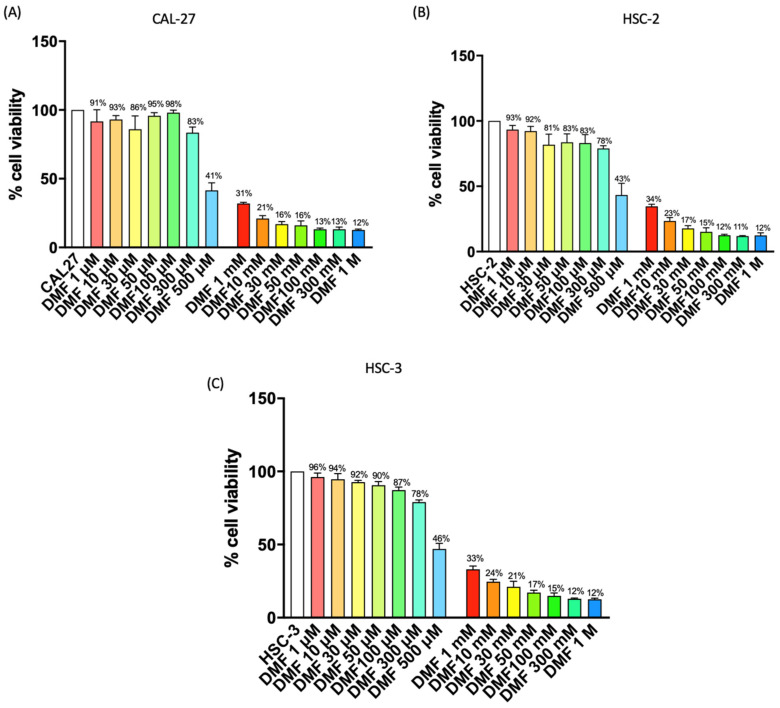
Effect of DMF on CAL27, HSC-2 and HSC-3 cell viability. DMF treatment at the concentrations of 500 μM was able to significantly reduce cell viability by 41% on CAL27 cells (**A**), 43% on HSC-2 (**B**) cells and 46% on HSC-3 cells (**C**). DMF 1 mM was able to reduce cell viability by 31% on CAL27 cells, 34% on HSC-2 cells and 33% on HSC-3 cells. DMF 10 mM reduced cell viability by 21% on CAL27 cells, 23% on HSC-2 cells and 24% on HSC-3 cells.

MTT assay was used to assess CAL27, HSC-2, and HSC-3 cell viability following 24 h of treatment with DMF at different concentrations (1 μM, 10 μM, 30 μM, 50 μM, 100 μM, 300 μM, 500 μM, 1 mM, 10 mM, 30 mM, 50 mM, 100 mM, 300 mM, 500 mM and 1 M). Our results show that DMF treatment at the concentrations of 1 μM, 10 μM, 30 μM, 50 μM, 100 μM, 300 μM it was unable to decrease cells viability enough. DMF at the concentrations of 500 μM was able to significantly reduce cell viability, respectively, by 41% in CAL27, 43% in HSC-2 and 46% in HSC-3; a further significant decrease in cell viability was obtained with treatment at the concentration of DMF 1 mM (respectively, of 31,34 and 33%). Even a DMF concentration of 10 mM contributed to an increase in cell cytotoxicity around 20%, as shown in Figure 1. Based on the MTT results, we decided to investigate in further analysis only DMF at concentrations of 500 μM, 1 mM and 10 mM because these represented the most cytotoxic concentrations. Furthermore, DMF showed comparable effects on cell viability in all three cell lines used; for this reason, we decided to continue to investigate its effect only on the CAL27 cell line, because it is representative and widely used as an in vitro model of OSCC.

#### 2.1.2. DMF Up-regulated Antioxidant Expression of HO-1 and MnSOD

**Figure 2:**
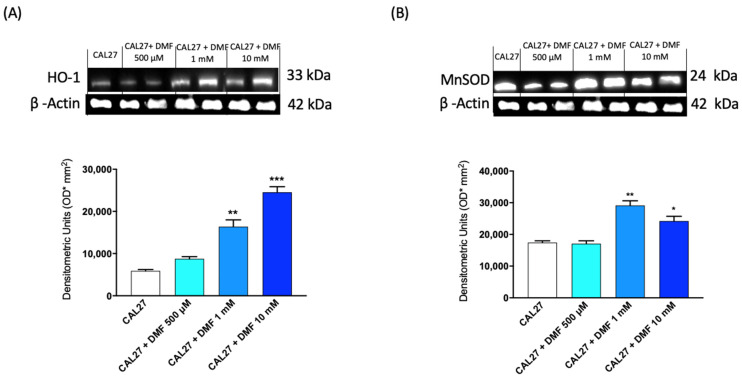
Effect of DMF on HO-1 and MnSOD expression in CAL27 cells. The blots revealed a significant increase of HO-1 expression following DMF treatment at the concentration of 1 mM and 10 mM (**A**). DMF at the same doses was also able to significant increase the expression of MnSOD (**B**). (**A**,**B**) * *p* < 0,05 vs. CAL27; ** *p* < 0.01 vs. CAL27; *** *p* < 0.001 vs. CAL27.

To evaluate the antioxidant response of DMF we analysed its effect on the expression of manganese superoxide dismutase (MnSOD) and heme oxygenase-1 (HO-1) by Western blot analysis. Our results showed that DMF treatment significantly up-regulated both HO-1 and MnSOD expression at the higher doses of 1 and 10 mM (Figure 2.).

#### 2.1.3. DMF Modulated Apoptosis Pathway

**Figure 3:**
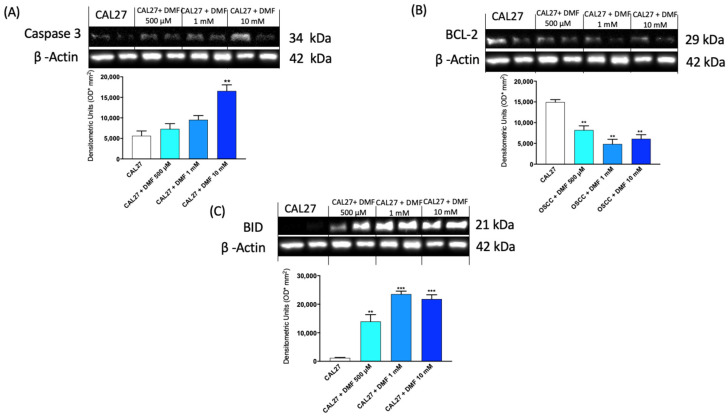
Effect of DMF on Caspase3, BID and BCL-2 expression in CAL27 cells (**A**–**C**). The blots revealed an increase of pro-apoptotic Caspase-3 and BID expression and a decrease of anti-apoptotic BCL-2 expression following DMF treatment at concentrations of 500 μM, 1 mM and 10 mM compared to CAL27 group. Data are representative of at least three independent experiments. (**A**,**B**) ** *p* < 0.01 vs. CAL27; (**C**) ** *p* < 0.01 vs. CAL27; *** *p* < 0.001 vs. CAL27.

In the valuation of the apoptotic pathway, the results obtained showed that DMF, at higher concentrations, significantly improved the levels of pro-apoptotic proteins Caspase 3 and BH3 interacting domain death agonist (BID) (Figure 3A–C), whereas the expression of the anti-apoptotic B-cell lymphoma 2 (BCL-2) protein was reduced after treatment with DMF already at the lowest concentration of 500 μM (Figure 3B).

#### 2.1.4. DMF Regulated EMT Markers

**Figure 4:**
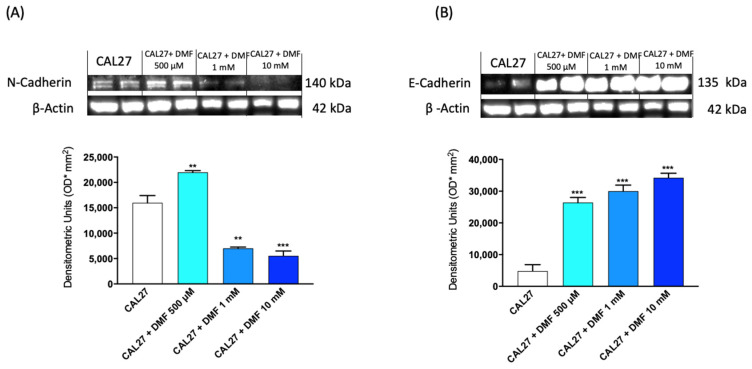
Effect of DMF on N-Cadherin and E-Cadherin expression in CAL27 cells. The blots revealed a significant decrease of N-Cadherin expression following DMF treatment at the concentration of 1 mM and 10 mM (**A**). DMF was also able to significant increase the expression of E-Cadherin at the concentration of 500 μM, 1 mM and 10 mM (**B**). (**A**,**B**) ** *p* < 0.01 vs. CAL27, *** *p* < 0.001 vs. CAL27.

Our results showed that DMF treatment led to a significant down-regulation of N-cadherin especially at concentrations of 1 mM and 10 mM (Figure 4A). The repression of E-cadherin, as previously mentioned, facilitates the epithelial to mesenchymal transition which leads to metastasis. Our results showed that DMF treatments up-regulates E-cadherin expression (Figure 4B), playing an important role in the maintenance of cell stability and suppression of cell proliferation.

#### 2.1.5. DMF Reduced OSCC Cell Migration

**Figure 5:**
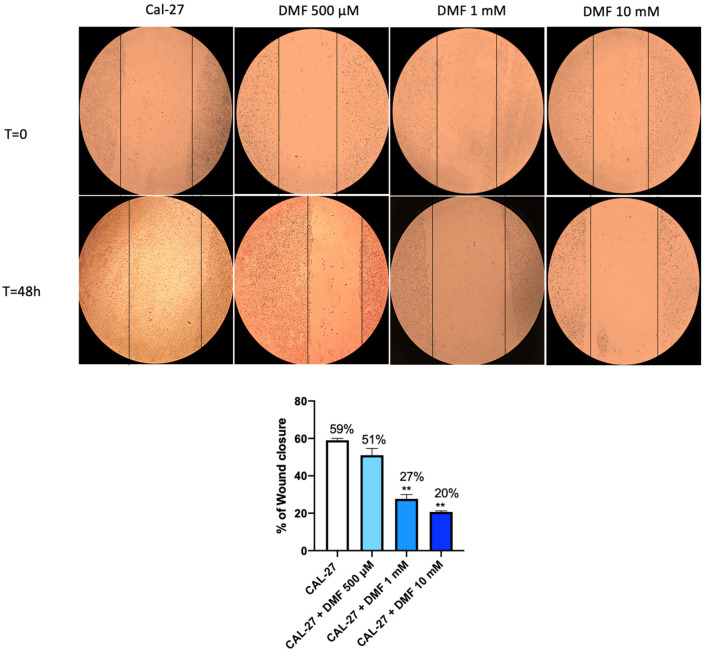
Effect of DMF on CAL27 migration. The wound healing assay (scratch test) revealed a significant reduction in the number of cells migrating to the scratched area, following 48 h of DMF treatment at the concentration of 1 mM and 10 mM. ** *p* < 0.01 vs. CAL27.

The effect of DMF on CAL27 cell migration was evaluated using an in vitro wound healing test. Confluent cells were scratched and then subjected to DMF treatment for 48 h. The images were acquired and the percentage of cells migrated to the scratched area was calculated. Our results showed that DMF led to a marked reduction in the number of cells migrating to the scratched area, particularly at the concentration of 1 mM and 10 mM after 48 h of treatment (Figure 5.).

#### 2.1.6. DMF Reduced TNFα Expression

**Figure 6:**
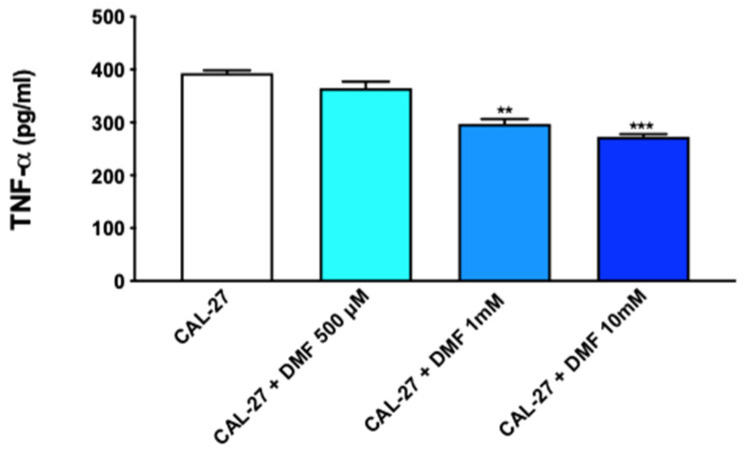
Effect of DMF on TNFα expression in CAL27 cell lysates. The enzyme-linked immunosorbent assay (ELISA) revealed a significant decrease of TNFα expression following DMF treatment at the concentration of 1 mM and 10 mM. ** *p* < 0.01 svs. CAL27, *** *p* < 0.001 vs. CAL27.

In order to interrogate the TNFα/TNFR1 signaling pathway, we utilized an enzyme-linked immunosorbent assay (ELISA), demonstrating that TNFα quantification was notably downregulated by DMF treatment in OSCC cell lysates in a dose-dependent way, as shown in Figure 6.

### 2.2. In Vivo Results

#### 2.2.1. DMF Reduced Tumor Growth on OSCC Orthotopic Model

**Figure 7:**
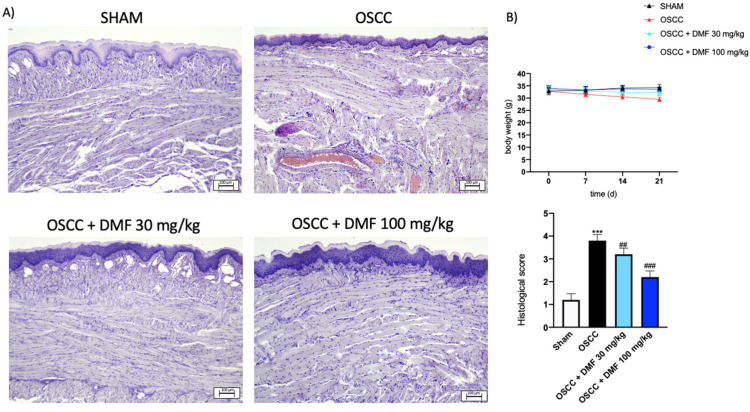
Effect of DMF on tumor growth in CAL27 orthotopic model. DMF treatment at doses of 30 and 100 mg/kg was able to significantly reduce submucosa tumor mass of the tongue and neutrophilic infiltration compared to control group (**A**). No important weight differences were detected between animals (**B**). Data are representative of at least three independent experiments. Sections were observed and photographed at 10× magnification. *** *p* < 0.001 vs. SHAM; ## *p* < 0.01 vs. OSCC; ### *p* < 0.001 vs. OSCC.

To evaluate the effect of DMF on the growth of OSCC cells in vivo, the CAL27 orthotopic model was established in nude mice. The histological analysis of the OSCC group showed a significant tumor mass growth, associated to an increase in necrosis and neutrophil infiltration compared to the sham group. In this context, treatment with DMF at doses of 30 and 100 mg/kg significantly reduced submucosa tumor mass of the tongue and neutrophilic infiltration in a dose-dependent manner (Figure 7A). Meanwhile, no important change in the animals’ body weight was shown (Figure 7B).

#### 2.2.2. DMF Confirmed the Modulation of Apoptotic and Antioxidant Pathways in the Orthotopic Model

**Figure 8:**
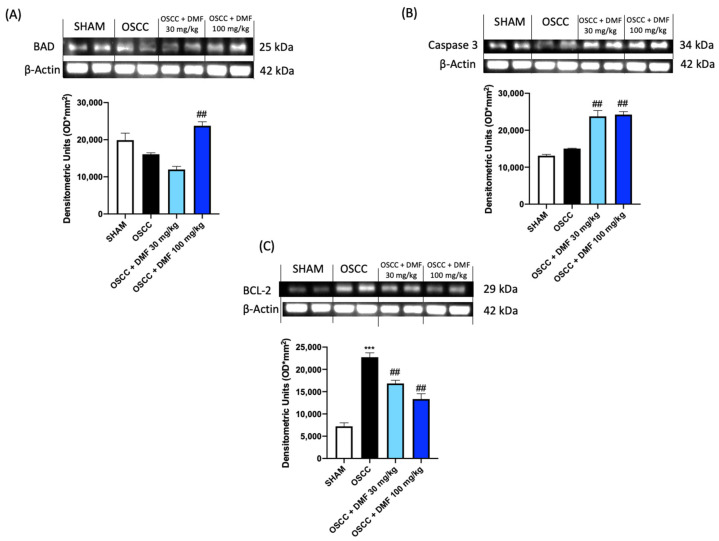
Effect of DMF on Caspase3, BAD and BCL-2 expression in tongue samples (**A**–**C**). The blots revealed an increase of pro-apoptotic BAD expression after DMF treatment especially at the dose of 100 mg/kg and Caspase 3 at both doses. A decrease of anti-apoptotic BCL-2 expression followed DMF treatment in a dose dependent manner compared to OSCC group. Data are representative of at least three independent experiments. (**A**,**B**) ## *p* < 0.01 vs. OSCC; (**C**) *** *p* < 0.001 vs. SHAM; ## *p* < 0.01 vs. OSCC.

**Figure 9:**
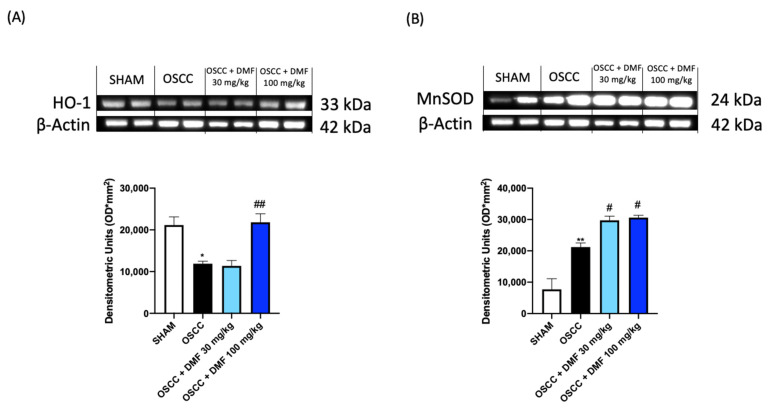
Effect of DMF on HO-1 and MnSOD expression in tongue samples (**A**,**B**). The blots revealed a significant increase of antioxidant expression of HO-1 after DMF treatment especially at the dose of 100 mg/kg and MnSOD at both doses. (**A**) * *p* < 0.05 vs. SHAM; ## *p* < 0.01 vs. OSCC; (**B**) ** *p* < 0.01 vs. SHAM; # *p* < 0.05 vs. OSCC.

To confirm the key role of apoptosis in the progression of OSCC, we evaluated some of the main apoptotic factors by Western blot analysis, also on the tongue samples collected in the OSCC orthotopic model. The results showed that DMF was able to significantly increase Caspase 3 and BCL2 associated agonist of cell death (BAD) expression and reduce BCL-2 expression, as shown in Figure 8. For the same reason, we also investigated the expression of antioxidant markers, demonstrating that DMF was able to significantly increase the expression of HO-1, only at the dose of 100 mg / kg, and of MnSOD, equally at both doses (Figure 9).

## 3. Discussion

OSCC is a common head and neck cancer characterized by a high incidence and mortality rate. Despite recent developments in the therapeutic management of OSCC, such as surgery, chemotherapy and radiotherapy, the prognosis of OSCC remains poor [20]. Currently, attention has been placed on the cellular mechanisms that favor neoplastic invasion and disease progression. At the cellular level, in fact, tobacco products such as polynuclear aromatic hydrocarbons (PAH) and nitrosamines exposes the epithelium of the oral mucosa to large quantities of ROS, including superoxide anion radicals (O⋅2), hydroxyl radicals (HO), hydroperoxyl (HO_2_), peroxyl (ROO⋅), alkoxyl (RO⋅) and hydrogen peroxide (H_2_O_2_), that are highly harmful and prevent the proper functioning of the physiological antioxidant mechanisms of the mucosa provided by antioxidants such as superoxide dismutase (SOD), HO-1, catalase (CAT), glutathione peroxidase (GPx), glutathione reductase (GRx), carotenes and vitamins [9]. Continuous and direct exposure to ROS is correlated to several cellular alterations including DNA strand breaks, membrane damage and mutations in tumor suppressor genes, factors underlying a condition called oxidative stress and directly involved in neoplastic transformation and progression of oral cancer [21]. Antioxidants are cytoprotective chemicals that prevent oxidative damage caused by free radicals and many studies revealed that DMF and its metabolite MMF promote cytoprotective activities by activating the antioxidant response of NRF2 pathway [22]. Our data revealed that DMF treatment enhanced the action of physiological antioxidant mechanisms by upregulating HO-1 and MnSOD expression both in vitro and in vivo models of OSCC.

The dysregulation of apoptotic mechanisms that promote programmed cell death plays a fundamental role in the progression of TSCC, favoring cell invasiveness and metastasis. Cancer cells can elude apoptotic mechanisms by becoming resistant to treatments and this results in therapeutic failure. Overexpression of BCL-2, an anti-apoptotic protein that plays a key role in cellular homeostasis, has been associated with the poor prognosis of OSCC [23]. Our data revealed that DMF was able to downregulate BCL-2 expression, by increasing the sensitivity of tumor cells to programmed cell death. BID is a p53 effector whose cleavage facilitates a conformational change in mitochondrial associated BAX, which functions promoting mitochondrial dysfunction and releasing cytochrome c [24]. In this study it was observed that DMF is able to upregulate the expression of BID, thus promoting apoptosis of OSCC cells. Caspase-3 is one of the main executioner caspases, which when cleaved and activated, degrades various cellular proteins and is responsible for morphological changes and DNA fragmentation in cells during apoptosis [25]. Our data showed a significant increase in Caspase-3 expression by inducing apoptosis in OSCC cells. These results were further confirmed in the OSCC orthotopic model, where DMF was able to downregulate the expression of BCL-2 and up-regulate the proapoptotic action of BAD and Caspase 3.

It has been widely suggested that the EMT program is activated in cancer cells, causing both the loss of certain epithelial characteristics such as apical–basal polarity, cell–cell junctions and basement membrane adherence, as well as the acquisition of some mesenchymal properties, which allow for migration and invasion.

Thus, tumor cells subjected to EMT show a series of molecular changes characterized by decreased expression of epithelial markers, as E-cadherin, ZO-1 and occludin, and increased expression of mesenchymal markers as N-cadherin, vimentin and fibronectin [26]. In this context, our results showed that DMF treatment led to significant downregulation of N-cadherin expression and upregulation of E-cadherin expression in OSCC cells, carrying out an important role in maintaining cell stability and reducing cell migration and invasion. Among the cytokines involved in the regulation of inflammatory processes and tumor promotion, TNFα plays a key role. Studies have shown that TNFα induces activation of the NF-κB pathway in OSCC cell lines, causing increased motility and invasiveness and inducing EMT in oral cancer cells, playing a key role in the development of metastases [27]. In this context, our data demonstrated that DMF significantly reduced TNFα expression in a dose-dependent manner, thus suggesting a protective role in reducing tumor migration.

Therefore, the results of the in vitro study, conducted on CAL27 cell lines, also supported by the data obtained in the OSCC orthotopic model, revealed that DMF was able to reduce the progression and growth of OSCC by modulating apoptosis and reducing oxidative stress and EMT. Therefore, DMF could be suggested as an alternative therapeutic strategy to counteract the progression of OSCC.

Although these preliminary results appear to be promising, further studies are needed to overcome some limitations. First, it would be interesting to evaluate the effect of DMF in combination with standard chemotherapy for OSCC, both in vitro and in vivo, in order to subsequently consider a possible phase 1 clinical study. Therefore, it would be important to evaluate through a dose-escalation study, if the current dose of 240 mg twice daily, approved for MS, demonstrates an acceptable safety and toxicity profile in combination with conventional therapy in OSCC patients.

## 4. Materials and Methods

### 4.1. In Vitro Studies

#### 4.1.1. Materials

DMF was obtained from Sigma–Aldrich Company (Milan, Italy). All chemicals were of the highest commercial grade available. All stock solutions were made in nonpyrogenic saline (0.9% NaCl; Baxter Healthcare Ltd., Thetford, Norfolk, UK) or 10% ethanol (Sigma–Aldrich).

#### 4.1.2. Cell Cultures

Human OSCC cell lines CAL27, HSC-2 and HSC-3 were obtained from ATCC American Type Culture Collection, Rockville, MD, USA). CAL27 cells were cultured in Dulbecco’s Modified Eagle Medium (DMEM) (Life Technologies, Gibco^®^; Carlsbad, CA, USA) supplemented with 10% fetal bovine serum (FBS, Life Technologies, Gibco^®^; Carlsbad, CA, USA), 100 U/mL of penicillin and 100 μg/mL of streptomycin. HSC-2 and HSC-3 cells were cultured in Medium Essential Eagle’s Medium (Sigma-Aldrich) supplemented with 10% fetal bovine serum (FBS) (Cultilab^®^, Campinas, Brazil), 100 U / mL penicillin and 100 μg/mL of streptomycin (Sigma-Aldrich, St. Louis, MO, USA). All cell lines were maintained in incubators at 37 ° C with 5% CO_2_.

#### 4.1.3. Cell Viability (MTT Assay)

Cell viability of CAL27, HSC-2 and HSC-3 cells was evaluated using a mitochondria-dependent dye for live cells (tetrazolium dye; MTT) (M5655; Sigma-Aldrich). CAL27, HSC-2 and HSC-3 cells were plated on 96-well plates at a density of 4 × 10^4^ cells/well to a final volume of 150 μL. After 24 h, CAL27, HSC-2 and HSC-3 cells were treated with DMF (Sigma-Aldrich^®^) for 24 h at increasing concentrations 1 μM, 10 μM, 30 μM, 50 μM, 100 μM, 300 μM, 500 μM, 1 mM, 10 mM, 30 mM, 50 mM, 100 mM, 300 mM, 500 mM and 1 M dissolved in basal medium. After 24 h cell were incubated at 37 °C with MTT (0.2 mg/mL) for 1 h, the medium was removed by aspiration and then cells were lysed with DMSO (100 μL). The extent of reduction of MTT to formazan was quantified by measurement of optical density at 540 nm (OD540) with a microplate reader, as previously described [28].

#### 4.1.4. Experimental Groups

1. Control group: OSCC cell lines CAL27, HSC-2 and HSC-3;
2. DMF 500 μM group: CAL27, HSC-2 and HSC-3 cells were treated with DMF 500 μM for 24 h;
3. DMF 1 mM group: CAL27, HSC-2 and HSC-3 cells were treated with DMF 1 mM group for 24 h;
4. DMF 10 mM group: CAL27, HSC-2 and HSC-3 cells were treated with DMF 10 mM for 24 h;

For other analysis, we continued to analyze only DMF 500 μM, 1 mM and 10 mM because represented the most cytotoxic concentrations revealed by MTT assay. Moreover, since DMF showed similar effects on cell viability in all three cell lines, we decided to continue to analyze the effect of DMF only on the CAL27 cell line, because it represents one of the most frequently used cell lines in the field of OSCC.

#### 4.1.5. Western Blot Analysis 

Western blot analysis was performed as previously described [29]. For cell lysates, CAL27 cells were washed twice with ice-cold phosphate buffered saline (PBS), collected and resuspended in lysis buffer containing 20 mM Tris-HCl pH 7.5, 10 mM NaF, 150 μL of NaCl, 1% Nonidet P-40 and protease cocktail of inhibitors (Catalog No. 11836153001; Roche, Switzerland). After 40 min, cell lysates were centrifuged at 12,000 rpm for 15 min at 4 ° C. Protein concentration was estimated using the Bio-Rad protein assay (Bio-Rad Laboratories, Hercules, CA, USA) using bovine serum albumin as a standard. The samples were then heated to 95 ° C for 5 min and equal amounts of proteins were separated by 10% -15% sodium dodecyl sulfate-polyacrylamide gel electrophoresis (SDS-PAGE) and transferred to a membrane of polyvinylidene difluoride (PVDF) (Immobilon-P, catalog # 88018; ThermoFisher Scientific). The following primary antibodies were used: anti-HO-1 (1: 500; sc-10789; Santa Cruz Biotechnology), anti-MnSOD (1:500; 06-984; Merck Millipore), anti-Casaspase 3 (1: 500; sc-7272; Santa Cruz Biotechnology), anti-BCL-2 (1: 500; sc-7382; Santa Cruz Biotechnology), anti-BID (1:500; sc-11423; Santa Cruz Biotechnology), anti-N-cadherin (1:500; #4061 Cell Signaling), anti-E-cadherin (1: 500; sc-8426, Santa Cruz Biotechnology). The antibody dilutions were made in PBS / 5% w / v skimmed milk powder / 0.1% Tween-20 (PMT) and the membranes were incubated overnight at 4° C. The membranes were then incubated with a secondary antibody (1: 2000; Jackson ImmunoResearch, West Grove, PA, USA) for 1 h at room temperature. To ensure that the stains were loaded with equal amounts of protein lysate, they were also incubated with β-actin antibody (cytosolic fraction 1: 1000; sc-47778; Santa Cruz Biotechnology) or foil A/C (nuclear fraction 1: 500; sc -376248; Santa Cruz Biotechnology). The signals were detected with enhanced chemiluminescence (ECL) detection system mixture (Thermo Fisher, Waltham, MA, USA).

#### 4.1.6. Wound Healing Assay (Scratch Test)

The effects of DMF on CAL27 cell migration was performed by the wound healing assay (scratch test), as previously described [30]. Briefly, 2 × 10^6^ CAL27 cells were plated on 60 mm plates (Corning Cell Culture, Tewksuby, MA, USA) in a final volume of 2 mL to obtain a confluent monolayer. At 24 h later, the cell monolayer was scratched, creating a straight line using a p200 pipette tip. After removing debris from each plate, cells were treated with increasing concentrations of DMF (500 μM, 1 mM, 10 mM and 50 mM) for 48 h. In the control group, however, normal culture medium was used. Finally, to record the wound width and therefore the migratory ability of the cells, photos of each plate were acquired through a phase contrast microscope at 0, 24 and 48 h. Cell migration rate was analyzed and calculated using Image J 1.53a software.

#### 4.1.7. Enzyme-Linked Immunosorbent Assay (ELISA) for TNFα

To evaluate the inflammatory response, the level of TNFα (Human TNF-alpha assay kit RAB1089 Sigma-Aldrich) was measured in cell lysates collected by enzyme-linked immunosorbent assay (ELISA), according to the manufacturer’s instructions. Briefly, 100 µL of standards and cell lysates were added to the appropriate wells and incubated for 2.5 h at room temperature. The solution was then discarded, and 4 washes were performed with 1× Wash Solution. Then, 100 µL of 1× Detection Antibody was added to each well and the plate was incubated for 1 h at room temperature. After repeated washes, 100 µL of streptavidin solution was added to each well and the plate was incubated for other 45 min. Finally, 100 µL of TMB One-Step Substrate Reagent was added to each well and the plate was incubated for 30 min protecting it from light. After adding 50 µL of Stop Solution to each well, the absorbance was read immediately using microplate reader at 450 nm [31].

### 4.2. In Vivo Studies

#### 4.2.1. Animals

For in vivo studies, the BALB/c nude male mice (25–30 g; 6–8 weeks of age) were used and purchased from Envigo (Milan, Italy). Animals were placed in a controlled environment and were fed with a standard diet and water ad libitum under pathogen-free conditions with a 12 h light/12 h dark. Animal study was approved by the University of Messina (n◦ 368/2019-PR released on 14 May 2019) in accordance with Italian regulations on the use of animals (D.M.116192) and Council Regulation regulations (EEC) (O.J. of E.C. L 358/1 12/18/1986).

#### 4.2.2. Orthotopic model of OSCC

For the orthotopic model 1.5 × 10^6^ CAL27 cells suspended in 50 μL of saline were injected into the left side of the submucosa of the tongue using an insulin syringe with a 28 G 1/2 needle, after anesthetizing the animals with 3% isoflurane. After the procedure, mice were fed with a soft food diet to reduce discomfort, monitored daily, and weighed periodically to assess overall health. Approximately three weeks after tumor inoculation, mice were treated with oral administrations of DMF every 3 days, at a dose of 30 mg/kg and 100 mg/kg, dissolved in physiological solution carried out every other day. At the end of the experiment, the animals were sacrificed through an overdose of anesthetic [32].

Mice were randomly divided into five experimental groups. Each group consisted of 8 mice, as described below:

1. SHAM group (8): oral administration of saline; saline
2. OSCC group (8): mice that received inoculation, orally administrated with saline;
3. OSCC + DMF 30 mg/kg group (8): mice that received tumor cells inoculation, orally administered with DMF at the dose of 30 mg/kg;
4. OSCC + DMF 100 mg/kg group (8): mice that received tumor cells inoculation, orally administered with DMF at the dose of 100 mg/kg.

#### 4.2.3. Histological Evaluation

Histological evaluation was performed as previously described [29]. Tongue samples were quickly removed and fixed with 10% buffered formalin for at least 24 h at room temperature. After dehydration in graded ethanol and xylene, tumor samples were embedded in paraffin and sectioned at 7 μm thickness. After staining with hematoxylin and eosin, sections were observed by an optical microscope (Axostar Plus equipped with Axio-Cam MRc, Zeiss, GE, Germany). The histological results are shown at 10× magnification (bar scale at 100 μm). All histological analyses were executed in a blinded manner.

#### 4.2.4. Western Blot Analysis 

Protein levels in tongue samples were quantified as previously described [ref]. Cytosolic proteins were prepared and separated electrophoretically to be transferred to nitrocellulose membranes. Membranes were blocked with 5% (*w*/*v*) dried nonfat milk in buffered saline (PM) for 45 min at room temperature and subsequently probed with specific antibodies: anti-HO-1 (1: 500; sc-10789; Santa Cruz Biotechnology), anti-MnSOD (1: 500; 06-984; Merck Millipore), anti-Caspase 3 (1: 500; sc-7272; Santa Cruz Biotechnology), anti-BCL-2 (1: 500; sc-7382; Santa Cruz Biotechnology), anti-BAD (1:500; sc-8044; Santa Cruz Biotechnology) in 1× PBS, 5% *w*/*v* dried nonfat milk and 0.1% Tween-20 (PMT) at 4 °C overnight. Membranes were incubated with peroxidase-conjugated goat anti-mouse IgG secondary antibody (1:2000, Jackson ImmunoResearch, West Grove, PA, USA) or peroxidase-conjugated goat anti-rabbit IgG secondary antibody (1:5000, Jackson ImmunoResearch, West Grove, PA, USA) for 1 h at room temperature. To establish that blots were loaded with equal amounts of proteins, they were also incubated in the presence of the antibody against β-actin protein (sc-8432, 1:500; Santa Cruz Biotechnology, Dallas, TX, USA). Signals were revealed with an enhanced chemiluminescence (ECL) detection system reagent according to the manufacturer’s instructions (Thermo, Waltham, MO, USA, cat# 457). The relative expression of protein bands was quantified by densitometry with Bio-Rad ChemiDoc XRS+ 6.1.0 software and standardized to β-actin levels as an internal control.

#### 4.2.5. Statistical Analysis

All values are expressed as mean  ±  standard deviation (SD) of N observations. Each analysis was performed three times with three samples replicates for each one. The results were analyzed by one-way analysis of variance (ANOVA) followed by a Bonferroni post hoc test for multiple comparisons. A value of *p* < 0.05 was considered significant.

## 5. Conclusions

In conclusion, the results obtained demonstrated that DMF treatment was able to modulate oxidative stress, apoptosis and EMT, offering new insights into their roles in the pathogenesis of oral cancer. Although further studies are needed to validate these preliminary data, DMF could be evaluated as a possible therapeutic strategy to counteract the growth of oral cancer through modulation of the pathways involved in oxidative stress and apoptosis.

## Data Availability

The data presented in this study are available on request from the corresponding author.
